# Usefulness of the ratio of brachial pre-ejection period to brachial ejection time in prediction of cardiovascular and overall mortality in patients with acute myocardial infarction

**DOI:** 10.1371/journal.pone.0245860

**Published:** 2021-01-29

**Authors:** Ho-Ming Su, Wen-Hsien Lee, Wei-Chung Tsai, Ying-Chih Chen, Nai-Yu Chi, Ching-Tang Chang, Chun-Yuan Chu, Tsung-Hsien Lin, Wen-Ter Lai, Sheng-Hsiung Sheu, Po-Chao Hsu

**Affiliations:** 1 Division of Cardiology, Department of Internal Medicine, Kaohsiung Medical University Hospital, Kaohsiung, Taiwan; 2 Department of Internal Medicine, Kaohsiung Municipal Siaogang Hospital, Kaohsiung Medical University, Kaohsiung, Taiwan; 3 Faculty of Medicine, College of Medicine, Kaohsiung Medical University, Kaohsiung, Taiwan; Kurume University School of Medicine, JAPAN

## Abstract

Left ventricular systolic function is a good indicator of cardiac function and a powerful predictor of adverse cardiovascular (CV) outcomes. High ratio of pre-ejection period (PEP) to ejection time (ET) is associated with reduced left ventricular systolic function. Brachial PEP (bPEP) and brachial ET (bET) can be automatically calculated from an ankle-brachial index (ABI)-form device and bPEP/bET was recently reported to be a new and useful parameter of cardiac performance. However, there were no studies evaluating the utility of bPEP/bET for prediction of CV and overall mortality in patients with acute myocardial infarction (AMI). We included 139 cases of AMI admitted to our cardiac care unit consecutively. ABI, bPEP, and bET were obtained from the ABI-form device within the 24 hours of admission. There were 87 overall and 22 CV mortality and the median follow-up to mortality event was 98 months. After multivariable analysis, high bPEP/bET was not only associated with increased long-term CV mortality (hazard ratio (HR) = 1.046; 95% confidence interval (CI): 1.005–1.088; P = 0.029), but also associated with long-term overall mortality (HR = 1.023; 95% CI: 1.001–1.045; P = 0.042). In addition, age was also a significant predictor for CV and overall mortality after the multivariable analysis. In conclusion, bPEP/bET was shown to be a significant predictor for CV and overall mortality in AMI patients after multivariable analysis. Therefore, by means of this novel parameter, we could easily find out the high-risk AMI patients with increased CV and overall mortality.

## Introduction

Acute myocardial infarction (AMI) is a cardiovascular (CV) emergency complicated with high morbidity and mortality [1]. Although evidence-based medication and advance of percutaneous coronary intervention improve the outcome of AMI, CV disease including AMI is still the leading cause of death in the world [2,3].

Left ventricular systolic function is a good indicator of cardiac function and a powerful predictor of adverse CV outcomes [4–8]. Because heart function impairment usually prolongs pre-ejection period (PEP) and shortens ejection time (ET), an increase in the ratio of PEP to ET is associated with reduced left ventricular systolic function. A high correlation between PEP/ET and left ventricular ejection fraction (LVEF) has been shown in patients with a wide variety of heart disease [9]. Brachial PEP (bPEP) and brachial ET (bET) can be automatically determined from an ankle-brachial index (ABI)-form device [10] and bPEP/bET was recently reported to be a new and useful parameter of systolic function and cardiac performance [11–13]. In addition, bPEP/bET was also reported to be associated with CV and overall mortality in the patients with chronic kidney disease and hemodialysis [12,13]. However, there were no studies evaluating the novel parameter of bPEP/bET for prediction of long-term CV and overall mortality in AMI patients. Therefore, our study was aimed to evaluate the issue.

## Materials and methods

### Study design and population

Patients with AMI admitted to the cardiac care unit from November 2003 to September 2004 were enrolled in our observational cohort study. Our inclusion criteria were patients with age ≥ 20 years old and type 1 AMI according to the universal definition of myocardial infarction [14]. AMI caused by atherothrombotic coronary artery disease and precipitated by atherosclerotic plaque disruption was designated as type 1 AMI. Patients with amputation of extremities, atrial fibrillation, and missing data of bPEP/bET were excluded. We finally enrolled 139 AMI patients. Because CV and overall mortality were our end points, we collected data of mortality up to December 2018. These data were acquired from the Collaboration Center of Health Information Application (CCHIA), Ministry of Health and Welfare, Taiwan. This study was approved by the institutional review board (IRB) committee in our hospital. The full name of our committee was IRB of Kaohsiung Medical University Hospital (IRB-KMUH). The reference number was KMU-IRB:93–147. We acquired informed consents from the patients and conducted our study according to the declaration of Helsinki. Demographic and medical data including age, gender, body mass index, percutaneous coronary intervention (PCI) or not, and comorbid conditions, such as dyslipidemia, diabetes, hypertension, ST segment elevation myocardial infarction (STEMI), and non-ST segment elevation myocardial infarction (NSTEMI) were obtained from medical records.

### Measurement of ABI, bPEP, bET, and blood pressures

**Fig 1:**
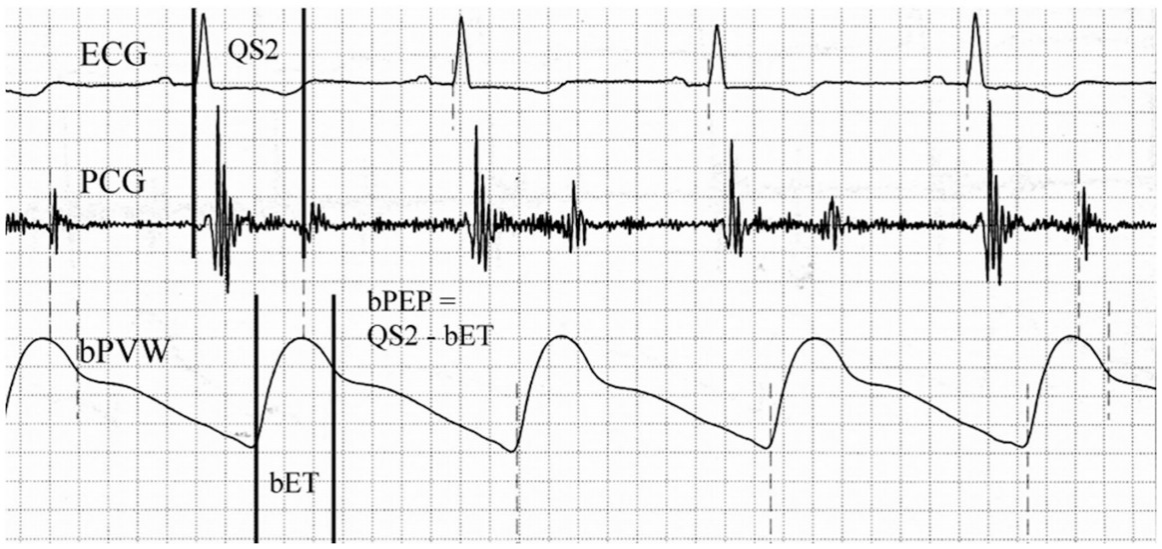
Brachial ejection time (bET) was automatically measured from the foot to the dicrotic notch of the brachial pulse volume waveform (bPVW). Total electromechanical systolic interval (QS2) was measured from the onset of the QRS complex on the electrocardiogram (ECG) to the first high-frequency vibrations of the aortic component of the second heart sound on the phonocardiogram (PCG). The brachial pre-ejection period (bPEP) was also automatically calculated by subtracting the bET from the QS2.

We measured ABI, bPEP, bET, and blood pressures by ABI-form device. This device could automatically measure blood pressures of extremities by oscillometric method [10,15] and it could also obtained the data of ABI, bPEP, and bET at the same time. The methods of measuring ABI, bPEP, and bET were the same as our previous study [16] (Fig 1). We performed above examination in every enrolled patient within twenty-four hours after admission.

### Definition of CV and overall mortality

All study participants were followed up till December 2018. Survival information and causes of death were obtained from the official death certificate and final confirmation by the Ministry of Health and Welfare. The causes of death were classified by the International Classification of Diseases 10th Revision. Causes of CV mortality were defined deaths due to hypertensive disease, cardiac disease, cerebral vascular disease, ischemic heart disease, myocardial infarction, heart failure, valvular heart disease, and atherosclerotic vascular disease. No participants lost follow-up in our study.

### Statistical analysis

We used SPSS 22.0 software (SPSS, Chicago, IL, USA) to perform the statistical analyses. Our data were shown as median (25th–75th percentile) for follow-up period or mean ± standard deviation, and percentage. Categorical variables were compared by Chi-square test. The significant variables and variables with significant trend in univariable analysis were selected for multivariable analysis. Youden index was used to find out the best cutoff value of bPEP/bET for the prediction of CV mortality. Continuous variables were compared by independent samples t-test. Multiple variables and time to mortality were adjusted by Cox proportional hazards model. P < 0.05 was regarded as significant.

## Results

Among the all subjects in our study, there were 30 patients with STEMI and 109 patients with NSTEMI. Fifty patients received PCI and 89 patients received medical treatment. Mean age was 63.8 ± 13.6 years. Median follow-up to mortality event was 98 months (25th–75th percentile: 17–175 months). There were 22 CV death and 87 all-cause death. In addition, we found that the best cutoff value of bPEP/bET for CV mortality prediction by receiver operating characteristic curve was 0.425.

Table 1 shows the characteristics between the patients with bPEP/bET below and above 0.425. Although there was no significant difference between the two groups, the patients with bPEP/bET ≥ 0.425 had a trend to have less male gender (P = 0.064) and lower AMI (P = 0.053).

**Table 1:** Baseline characteristics of the study population classified by bPEP/bET below and above 0.425.

Baseline Characteristics	bPEP/bET < 0.425	bPEP/bET ≥ 0.425	P value
Number	95	44	
Age (yr)	63 ± 14	65 ± 12	0.410
Male gender (%)	78.9%	63.6%	0.064
Dyslipidemia (%)	35.8%	22.7%	0.170
Diabetes mellitus (%)	26.3%	20.5%	0.529
Hypertension (%)	43.2%	43.2%	1.000
STEMI (%)	24.2%	15.9%	0.376
NSTEMI (%)	75.8%	84.1%	0.376
PCI (%)	40.0%	27.3%	0.184
Body mass index	24.7 ± 3.7	23.8 ± 3.7	0.202
Ankle brachial index	0.99 ± 0.18	0.93 ± 0.19	0.053

Abbreviations: bET, brachial ejection time; bPEP, brachial pre-ejection period; NSTEMI, non-ST segment elevation myocardial infarction; PCI, percutaneous coronary intervention; STEMI, ST segment elevation myocardial infarction.

**Fig 2:**
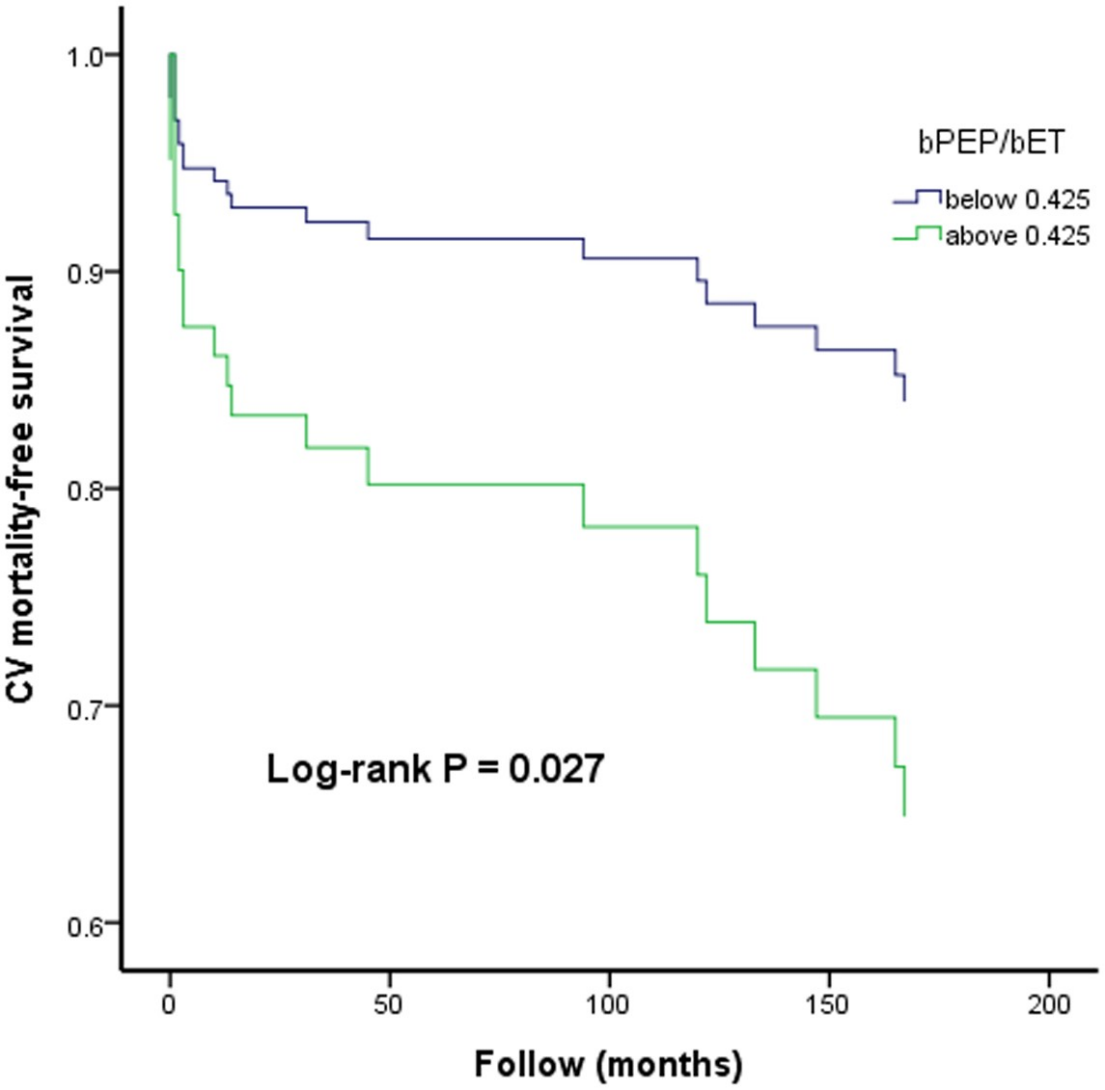
The Kaplan-Meier curve for cardiovascular (CV) mortality-free survival subdivided according to bPEP/bET below and above 0.425.

Fig 2 illustrates the Kaplan-Meier curves for CV mortality-free survival subdivided according to bPEP/bET below and above 0.425 (log-rank P = 0.027).

Table 2 shows the characteristics between the patients with STEMI and NSTEMI. Patients with STEMI had higher percentage of PCI. However, no significant difference was found in the other variables.

**Table 2:** Baseline characteristics of the study population classified by STEMI and NSTEMI.

Baseline Characteristics	STEMI	NSTEMI	P value
Number	30	109	
Age (yr)	64 ± 17	64 ± 13	0.963
Male gender (%)	73.3%	74.3%	1.000
Dyslipidemia (%)	36.7%	30.3%	0.513
Diabetes mellitus (%)	26.7%	23.9%	0.811
Hypertension (%)	40.4%	53.3%	0.219
PCI (%)	96.7%	19.3%	<0.001
Body mass index	24.2 ± 3.8	24.5 ± 3.7	0.731
Ankle brachial index	1.01 ± 0.21	0.97 ± 0.17	0.288
bPEP/bET	0.36 ± 0.07	0.39 ± 0.12	0.079
Cardiovascular mortality	10.0%	17.4%	0.408
Overall mortality	60.0%	63.3%	0.832

Abbreviations as in Table 1.

Table 3 reveals the predictors for CV mortality using Cox proportional hazards model. In our univariable analysis, age, ABI, and bPEP/bET were shown to predict CV mortality significantly and hypertension was shown to have a significant trend (P = 0.057). Therefore, we adjusted the age, hypertension, ABI, and bPEP/bET for multivariable analysis. After multivariable analysis, only age and bPEP/bET (hazard ratio [HR] = 1.046; 95% confidence interval [CI]: 1.005–1.088; P = 0.029) were significant predictors for CV mortality.

**Table 3:** Predictors of cardiovascular mortality using Cox proportional hazards model by univariable and multivariable analyses.

Parameter	Univariable analysis		Multivariable analysis	
	HR (95% CI)	*P*	HR (95% CI)	*P*
Age (per 1 year)	1.042(1.006–1.079)	<0.001	1.044(1.008–1.082)	0.015
Gender (Male vs Female)	0.752(0.293–1.929)	0.553	-	-
Diabetes mellitus (Yes vs No)	1.266(0.495–3.239)	0.622	-	-
Hypertension (Yes vs No)	2.292(0.977–5.378)	0.057	-	0.214
Dyslipidemia (Yes vs No)	1.298(0.544–3.095)	0.557	-	-
STEMI (Yes vs No)	0.548(0.162–1.853)	0.333	-	-
PCI (Yes vs No)	0.891(0.363–2.187)	0.801	-	-
Body mass index (per 1kg/m^2^)	0.978(0.867–1.103)	0.717	-	-
Lower ankle brachial index (Per 0.1)	0.798(0.654–0.974)	0.027	-	0.383
bPEP/bET (Per 0.01)	1.042(1.003–1.083)	0.037	1.046(1.005–1.088)	0.029

HR: Hazard ratio; CI: Confidence interval; other abbreviations as in Table 1.

Table 4 reveals the predictors for overall mortality using Cox proportional hazards model. In our univariable analysis, age, female gender, presence of hypertension, body mass index, and ABI were shown to predict overall mortality significantly. bPEP/bET had a trend to have significant finding (P = 0.087). Therefore, we adjusted the age, gender, hypertension, body mass index, ABI, and bPEP/bET for multivariable analysis. After multivariable analysis, only age and bPEP/bET (HR = 1.023; 95% CI: 1.001–1.045; P = 0.042) were significant predictors for overall mortality.

**Table 4:** Predictors of overall mortality using Cox proportional hazards model by univariable and multivariable analyses.

Parameter	Univariable analysis		Multivariable analysis	
	HR (95% CI)	*P*	HR (95% CI)	*P*
Age (per 1 year)	1.066(1.047–1.086)	<0.001	1.067(1.048–1.087)	<0.001
Gender (Male vs Female)	0.625(0.396–0.987)	0.044	-	0.417
Diabetes mellitus (Yes vs No)	1.101(0.678–1.788)	0.698	-	-
Hypertension (Yes vs No)	1.677(1.100–2.556)	0.016	-	0.999
Dyslipidemia (Yes vs No)	1.160(0.741–1.813)	0.516	-	-
STEMI (Yes vs No)	0.869(0.516–1.461)	0.596	-	-
PCI (Yes vs No)	1.110(0.717–1.719)	0.641	-	-
Body mass index (per 1kg/m^2^)	0.885(0.830–0.943)	<0.001	-	0.172
Lower ankle brachial index (Per 0.1)	0.808(0.731–0.894)	<0.001	-	0.996
bPEP/bET (Per 0.01)	1.018(0.997–1.039)	0.087	1.023(1.001–1.045)	0.042

HR: Hazard ratio; CI: Confidence interval; other abbreviations as in Table 1.

## Discussion

This study aimed to investigate the novel parameter of bPEP/bET for prediction of CV and overall mortality in patients with AMI. We had several major findings as below mentioned. First, high bPEP/bET was a significant predictor for CV mortality after multivariable analysis. Second, although bPEP/bET only had a trend to be associated with increased overall mortality in the univariable analysis, it became a significant predictor for overall mortality after multivariable analysis. Third, low ABI was not associated with CV and overall mortality after the multivariable analysis. Forth, bPEP/bET can be easily obtained from the ABI-form device without the necessity of echocardiography and the experts who are familiar with the echocardiography, it can be used as a simple and useful tool for mortality prediction in AMI patients.

AMI is an emergency of CV disease and a vital public health issue. Although the mortality rate of AMI gradually decreased in recent decades, it still complicated with relative high mortality [1]. Therefore, how to simply predict outcomes for AMI patients is very important because the treatment may be different for the different risk group patients.

A ABI-form device (VP 1000) could automatically measure blood pressures of four limbs by oscillometric method [10,15]. It could also acquire the values of ABI, bPEP, and bET at the same time [10]. We have recently found bPEP/bET has a significant correlation with LVEF and is a useful parameter in prediction of reduced left ventricular systolic function [11–13]. LVEF is a widely used parameter for the assessment of cardiac systolic function and has been established as an excellent predictor of adverse CV outcomes [4–8]. Chen SC et al. ever reported that bPEP/bET was a useful predictor for prediction of mortality in chronic kidney disease and hemodialysis patients [12,13]. Therefore, our study tried to investigate whether bPEP/bET was also a good parameter for prediction of long-term CV and overall mortality in AMI patients. In our study, bPEP/bET was associated with CV mortality not only in univariable analysis, but also in multivariable analysis. In addition, although bPEP/bET only showed a trend to be associated with overall mortality in univariable analysis, it became significant predictor of overall mortality after multivariable analysis. Therefore, screening AMI patients simply by means of bPEP/bET can help physicians to identify high risk group with increased mortality.

Low ABI calculated as the ratio of ankle systolic blood pressure to brachial systolic blood pressure is associated with an increased overall and CV mortality in different population, such as patients with diabetes, atrial fibrillation, ischemic heart disease, chronic kidney disease, and hemodialysis [17–21]. However, in our study, although ABI was a significant predictor for CV and overall mortality in the univariable analysis, it became insignificant after multivariable analysis. Therefore, bPEP/bET as an excellent parameter of cardiac performance might play more important role than ABI for mortality prediction in the patients with AMI.

In addition, overall mortality rate in our study was relative high (62.6%, 87/139). There were several possible reasons to explain the high mortality rate. First, our study enrolled the AMI patients about 16 to 17 years ago. Second, only 36% of the patients received PCI at that time. Third, our patients were relatively old with mean age 63.8 ± 13.6 years.

### Study limitations

We had some limitations in this study. First, echocardiographic parameters and medications were not adjusted in the multivariable analysis because of incomplete data. In our hospital, the patient chart was not available if he or she did not visit our hospital again more than 10 years. Although medications post AMI such as dual antiplatelet, beta blockers, angiotensin converting enzyme inhibitors, angiotensin receptor blockers, and statins might affect the long-term outcomes, we could not perform statistical analysis due to incomplete data of medications. Second, we did not perform myocardial perfusion scan to evaluate the infarction size in our current study and could not show the evidence that bPEP/bET was also correlated with the infarction size. Third, the sample size was relatively small in the study.

## Conclusions

Our study was the first study to investigate the parameter of bPEP/bET in AMI patients for prediction of long-term CV and overall mortality. After multivariable analysis, bPEP/bET, a surrogate parameter of cardiac performance, was shown to be an independent predictor for long-term CV and overall mortality in AMI patients Therefore, by means of this novel parameter, we could easily find out the high-risk AMI patients with increased CV and overall mortality.

## Supporting information

S1 Data AMI data (20201102). (CSV)
